# Activating Transcription Factor 3 Deficiency Promotes Cardiac Hypertrophy, Dysfunction, and Fibrosis Induced by Pressure Overload

**DOI:** 10.1371/journal.pone.0026744

**Published:** 2011-10-28

**Authors:** Heng Zhou, Di-Fei Shen, Zhou-Yan Bian, Jing Zong, Wei Deng, Yan Zhang, Yuan-Yuan Guo, Hongliang Li, Qi-Zhu Tang

**Affiliations:** 1 Department of Cardiology, Renmin Hospital of Wuhan University, Wuhan, China; 2 Cardiovascular Research Institute of Wuhan University, Wuhan, China; Northwestern University, United States of America

## Abstract

Activating transcription factor 3 (ATF3), which is encoded by an adaptive-response gene induced by various stimuli, plays an important role in the cardiovascular system. However, the effect of ATF3 on cardiac hypertrophy induced by a pathological stimulus has not been determined. Here, we investigated the effects of ATF3 deficiency on cardiac hypertrophy using *in vitro* and *in vivo* models. Aortic banding (AB) was performed to induce cardiac hypertrophy in mice. Cardiac hypertrophy was estimated by echocardiographic and hemodynamic measurements and by pathological and molecular analysis. ATF3 deficiency promoted cardiac hypertrophy, dysfunction and fibrosis after 4 weeks of AB compared to the wild type (WT) mice. Furthermore, enhanced activation of the MEK-ERK1/2 and JNK pathways was found in ATF3-knockout (KO) mice compared to WT mice. *In vitro* studies performed in cultured neonatal mouse cardiomyocytes confirmed that ATF3 deficiency promotes cardiomyocyte hypertrophy induced by angiotensin II, which was associated with the amplification of MEK-ERK1/2 and JNK signaling. Our results suggested that ATF3 plays a crucial role in the development of cardiac hypertrophy via negative regulation of the MEK-ERK1/2 and JNK pathways.

## Introduction

Cardiac hypertrophy occurs in response to stresses, such as pressure and volume overload, neurohormones and mutations in genes encoding sarcomeric proteins [1]. It can provide mechanical advantages that help maintaining normal ejection performance to endure increased workload [2]. However, in the long term, cardiac hypertrophy predisposes individuals to heart failure, arrhythmia and sudden death [3]. Although a series of studies have illustrated that signaling pathways, including mitogen activated protein kinases (MAPKs), phosphatidylinositol 3-kinase (PI3K)/AKT and calcineurin/nuclear factor of activated T cells (NFAT), play important roles in hypertrophic responses [3], the mechanisms that regulate these pathways have not been clearly elucidated. Therapies for cardiac hypertrophy still primarily focus on modulating hemodynamics; therefore, it is important to clarify the mechanisms involved in cardiac hypertrophy and to discover new antihypertrophic targets.

Activating transcription factor (ATF) 3 is a member of the activating transcription factor/cAMP responsive element-binding protein (ATF/CREB) family of transcription factors, which bind to a consensus DNA sequence (TGACGTCA) and share a leucine zipper (bZIP) element [4], [5], [6]. Members of this family bind to specific DNA via the basic region in this domain, and form homodimers or heterodimers with other bZIP-containing proteins via the leucine zipper region [4], [6]. ATF3 has a low expression level in quiescent cells, but is increased under stress conditions, such as injury, ischemia, ischemia/reperfusion or chemical toxin, and is considered as an adaptive-response gene [5], [7]. Signals including cytokines, chemokines, growth factors/hormones or DNA damage can induce ATF3 expression [5], [7], [8].

In the cardiovascular system, ATF3 expression is induced by TNF-α and acute hypoxia in vascular endothelial cells [9], [10]. A variety of stimuli, including serum, angiotensin II (Ang II) and H_2_O_2_, can increase the ATF3 expression in vascular smooth muscle cells (VSMCs), and, knockdown of ATF3 induces VSMCs apoptosis, caspase-3 cleavage and cytochrome c release [11]. In addition, doxorubicin-treated neonatal rat cardiomyocytes have high ATF3 expression via the JNK pathway, while ATF3 overexpression protects cardiomyocytes from doxorubicin-induced apoptosis [12]. *In vivo*, the cardiac expression of ATF3 is induced by ischemia/reperfusion [13] and Ang II [14], and transgenic mice with cardiac-specific expression of ATF3 exhibit atrial enlargement, atrial and ventricular hypertrophy, fibrosis, reduced contractility and aberrant conduction[13]. However, the effect of ATF3 deficiency on cardiac hypertrophy, especially, when induced by pathological stimuli, has not been determined. For this study, we used ATF3-knockout (KO) mice and cultured neonatal mouse cardiomyocytes from ATF3-KO mice to investigate the role of ATF3 in the hypertrophic response. We show that ATF3 deficiency in mice promotes cardiac hypertrophy, dysfunction and fibrosis in response to pressure overload, suggesting a crucial role for ATF3 in modulating cardiac remodeling.

## Materials and Methods

### Animals and animal models

All animal procedures were performed in accordance with the *Guide for the Care and Use of Laboratory Animals* published by the US National Institutes of Health (NIH Publication No. 85-23, revised 1996) and approved by the Animal Care and Use Committee of Renmin Hospital of Wuhan University (protocol number: 00013274). Male ATF3-KO mice (C57 background) and their wild-type (WT) littermates aged 8 to 10 weeks were used in the studies. The ATF3-KO mice were kindly provided by Dr. Tsonwin Hai from Department of Molecular and Cellular Biochemistry and Center for Molecular Neurobiology, Ohio State University, Columbus, Ohio, USA. Genotyping was performed by PCR as described previously [15]. Aortic banding (AB) was performed as described previously [16], [17]. Hearts and lungs of the sacrificed mice were harvested and weighed to compare heart weight/body weight (HW/BW, mg/g), lung weight/body weight (LW/BW, mg/g), and heart weight/tibia length (HW/TL, mg/mm) ratios in KO and WT mice.

### Echocardiography and hemodynamics

Echocardiography was performed in anesthetized (1.5% isoflurane) mice using a Mylab 30CV (ESAOTE S. P. A) with a 10-MHz linear array ultrasound transducer. The left ventricle (LV) dimensions were assessed in parasternal short-axis view. End-systole or end-diastole was defined as the phase in which the smallest or largest area of the LV was obtained, respectively.

For hemodynamic measurements, mice were anesthetized with 1.5% isoflurane, and a microtip catheter transducer (SPR-839, Millar Instruments, Houston, TX, USA) was inserted into the right carotid artery and advanced into the left ventricle. The signals were continuously recorded using a Millar Pressure-Volume System (MPVS-400, Millar Instruments, Houston, TX, USA), and the data were processed by PVAN data analysis software.

### Histological analysis and immunohistochemistry

Hearts were excised, washed with PBS, arrested in diastole with 10% KCl, weighed, fixed by perfusion with 10% formalin, and embedded in paraffin. Hearts were cut transversely close to the apex to visualize the left and right ventricles. Several sections of each heart (4–5 µm thick) were prepared, stained with hematoxylin and eosin (H&E) for histopathology or picrosirius red (PSR) for collagen deposition and then visualized by light microscopy. For myocyte cross-sectional area, the sections were stained with FITC-conjugated WGA (Invitrogen) to visualize membranes and DAPI to visualize nuclei. A single myocyte was measured with a quantitative digital image analysis system (Image Pro-Plus, version 6.0). The outline of 100 myocytes was traced in each group. For immunohistochemistry, the heart sections were heated using the pressure cooker method for antigen retrieval, incubated with anti-ATF3 (Santa Cruz Biotechnology, sc-188) and subsequently an anti-rabbit EnVisionTM+/HRP reagent, and stained using a DAB detection kit.

### Quantitative real-time RT-PCR

Real-time PCR was used to detect the mRNA expression levels of hypertrophic and fibrotic markers. Total RNA was extracted from frozen mouse cardiac tissue or cultured cardiac myocytes using TRIzol (Invitrogen, 15596-026), and their yields and purities were spectrophotometrically estimated using the A260/A280 and A230/260 ratios via a SmartSpec Plus Spectrophotometer (Bio-Rad). The RNA (2 µg of each sample) was reverse-transcribed into cDNA using oligo (DT) primers and the Transcriptor First Strand cDNA Synthesis Kit (Roche, 04896866001). The PCR amplifications were quantified using a LightCycler 480 SYBR Green 1 Master Mix (Roche, 04707516001) and the results were normalized against glyceraldehyde-3-phosphate dehydrogenase (GAPDH) gene expression.

### Western blotting

Cardiac tissue and cultured cardiac myocytes were lysed in RIPA lysis buffer, and the protein concentration was measured with the BCA protein assay kit (Themo, 23227) by ELISA (Synergy HT, Bio-tek). The cell lysate (50 ug) was used for SDS/PAGE, and the proteins were then transferred to Immobilon-FL transfer membranes (Millipore, IPFL00010). The primary antibodies included antibodies specific for p-MEK1/2 (Cell Signaling Technology, 9154), T-MEK1/2 (Cell Signaling Technology, 9122), p-ERK1/2 (Cell Signaling Technology, 4370), T-ERK1/2 (Cell Signaling Technology, 4695), p-P38 (Cell Signaling Technology, 4511), T-P38 (Cell Signaling Technology, 9212), p-JNK (Cell Signaling Technology, 4668), T-JNK (Cell Signaling Technology, 9258), p-p90RSK(Cell Signaling Technology, 9335), T-p90RSK (Cell Signaling Technology, 9347), p-AKT (Cell Signaling Technology, 4060), T-AKT (Cell Signaling Technology, 4691), GAPDH (Cell Signaling Technology, 2118), and ATF3 (Santa Cruz Biotechnology, sc-188). The secondary antibody was goat anti-rabbit (LI-COR, 926-32211) IgG. The blots were scanned by a two-color infrared imaging system (Odyssey, LI-COR). Specific protein expression levels were normalized to GAPDH protein for total cell lysates and cytosolic proteins.

### Neonatal mouse cardiomyocyte culture and surface area

Primary cultures of mouse ventricular cardiomyocytes were prepared as described previously [18] with minor modifications. Newborn (1–2-day old) WT and KO mice (C57 background) were killed by swift decapitation, and the ventricles were minced and digested in PBS (HyClone, SH30256.01B) containing 0.03% trypsin (HyClone, SH30042.01) and 0.04% collagenase type II (Sigma, C6885-1G). The harvested cells were centrifuged, and the sediment was resuspended in Dulbecco's modified Eagle's medium (DMEM)/F12 1∶1 medium (HyClone, SH30023.01B) supplemented with 20% fetal bovine serum (FBS; HyClone, SV30087.02), 100 U/ml penicillin/100 mg/ml streptomycin (Gibco, 15140) and 0.1 mmol/L bromodeoxyuridine (BrdU; Sigma, B5002). The fibroblast content of the cell suspension was removed by a differential attachment technique. Cell-rich medium was planted to 35-mm dishes coated with gelatin. After 48 h, the culture medium was changed to serum-free DMEM/F12 for 12 h before the experiment, and then, the cultured myocytes were stimulated with 1 µM Ang II (Sigma, A9525).

To identify the cardiomyocytes and assess cardiomyocyte hypertrophy, we characterized cells by immunocytochemistry for cardiac α-actinin. The cells were washed with PBS, fixed with RCL2 (ALPHELYS, RCL2-CS24L), permeabilized in 0.1% Triton X-100 in PBS, and stained with anti-α-actinin (Millipore, 05-384) at a dilution of 1∶100 in 1% goat serum. The secondary antibody was Alexa Fluor® 568 goat anti-mouse IgG (Invitrogen, A11004). The myocytes on coverslips were mounted onto glass slides with SlowFade Gold antifade reagent with DAPI (Invitrogen, S36939).

### Human heart samples

Samples of human failing hearts were collected from the left ventricles of dilated cardiomyopathy (DCM) patients undergoing heart transplants. Control Samples were obtained from the left ventricles of the normal heart donors. The samples were obtained with the approval of the local Ethical Committee (Renmin Hospital of Wuhan University Human Research Ethics Committee, Wuhan, China). The investigation conformed to the principles outlined in the Declaration of Helsinki. Informed written consent was obtained from all subjects.

### Statistical analysis

Data are expressed as the means ± SEM. Differences among the groups were determined by two-way ANOVA followed by a *post hoc* Tukey test. Comparisons between the two groups were performed by the unpaired Student's t-test. *P*<0.05 was considered to be significantly different.

## Results

### ATF3 expression in patients with dilated cardiomyopathy and the pressure overload-induced hypertrophic mouse model

**Figure 1:**
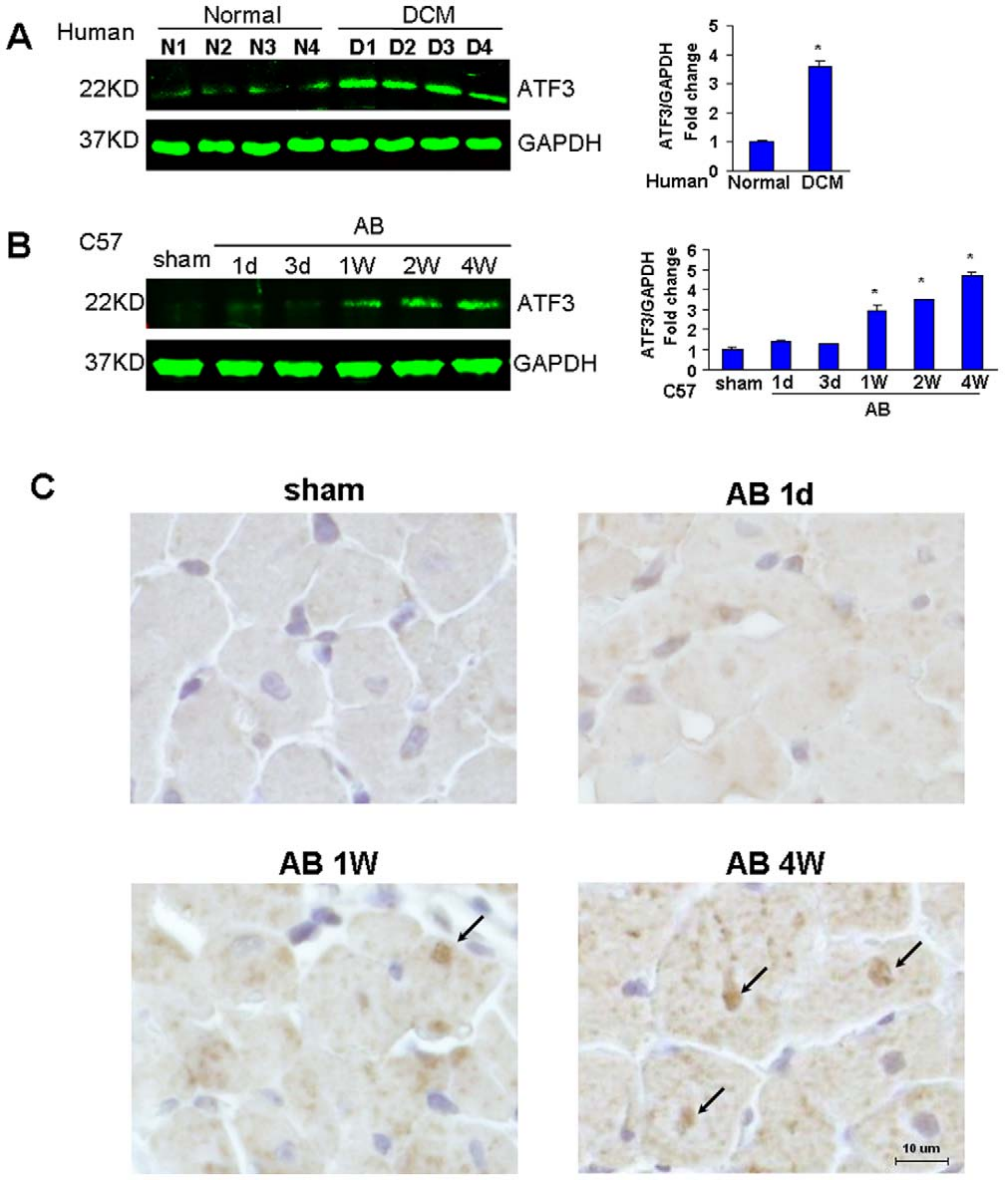
ATF3 expression in hypertrophic heart. A, Western blot analysis of cardiac expression of ATF3 in normal donators and in DCM patients (n = 4). *P<0.05 vs normal donators. B, Western blot analysis of cardiac ATF3 protein from WT mice after aortic banding at the time points indicated (n = 6). *P<0.05 vs sham. C, Immunohistochemistry of cardiac ATF3 protein from WT mice after aortic banding at time points indicated.

We compared the cardiac expression of ATF3 in DCM patients to that of healthy donators, and found that it was increased by 3.6-fold (Figure 1A). Then we examined ATF3 expression in response to pressure overload in mice. ATF3 expression Levels in the murine heart were gradually elevated from 1 day to 4 weeks after the AB operation (Figure 1B, C).

### ATF3 deficiency promotes cardiac hypertrophy and dysfunction in response to AB

**Figure 2:**
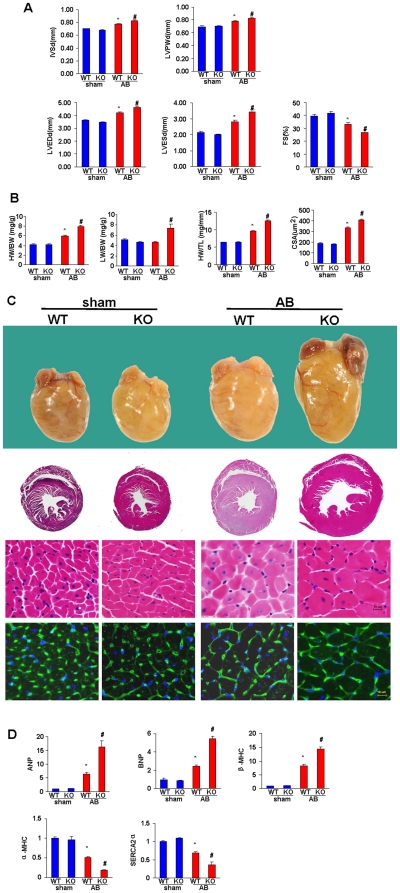
Effects of ATF3 on cardiac hypertrophy. A, Echocardiography results from 4 group mice at 4 weeks after AB or sham surgery (n = 8). B, Statistical results of the HW/BW, LW/BW, HW/TL and myocyte cross-sectional areas of indicated groups. C, Gross hearts, HE staining and WGA-FITC staining of sham and AB mice at 4 weeks post surgery. D, Expression of transcripts for ANP, BNP, β-MHC, α-MHC and SERCA2α induced by AB were determined by RT-PCR analysis (n = 6). *P<0.05 vs WT/sham. # *P*<0.05 vs WT/AB after AB.

To evaluate the effect of ATF3 on cardiac hypertrophy, we performed the AB surgery or a sham operation on ATF3-KO mice and WT littermates. After 4 weeks, echocardiography was performed to assess the chamber diameter, wall thickness and function of the left ventricle. There were no significant changes between the sham-operated KO and WT mice; however, KO mice exhibited deteriorated cardiac hypertrophy and dysfunction compared to WT mice, as measured by LVEDD, LVESD, interventricular septal thickness at end-diastole (IVSD), left ventricular posterior wall thickness at end-diastole (LVPWD), and fractional shortening (FS) after 4 weeks of AB (Figure 2A). Pressure-volume (PV) loop analysis further revealed the exacerbated hemodynamic dysfunction of the LV in ATF3-KO mice, as measured by parameters that reflect the volume, systolic function and diastolic function of LV (Table 1). The HW/BW, HW/TL and LW/BW ratios and the cardiomyocyte cross-sectional area (CSA) were also strikingly increased in the pressure-overloaded KO mice compared to the WT mice (Figure 2B). The gross hearts, H&E staining and WGA staining confirmed the adverse effect of ATF3 deficiency on cardiac remodeling (Figure 2C). In addition, we used real-time PCR analysis to examine the mRNA expression of markers of cardiac hypertrophy, including atrial natriuretic peptide (ANP), B-type natriuretic peptide (BNP), β-myosin heavy chain (β-MHC), α-myosin heavy chain (α-MHC) and sarcoendoplasmic reticulum Ca^2+^-ATPase (SERCA2α). AB-induced up-regulation of cardiac fetal genes including ANP, BNP and β-MHC were greater in ATF3-KO mice, which was accompanied with the down-regulation of α-MHC and SERCA2α (Figure 2D). These results suggested that ATF3 deficiency promotes cardiac hypertrophy and deteriorates impaired cardiac function after pressure overload.

**Table 1:** Hemodynamic parameters in mice after 4 weeks of surgery.

Parameter	Sham		AB	
	WT(n = 6)	KO(n = 6)	WT(n = 6)	KO(n = 6)
P–V loop analysis				
HR (min^−1^)	479±8	460±8	461±11	460±10
ESP (mmHg)	108.2±2.3	110.8±2.5	152.7±4.9*	155.4±7.8*
EDP (mmHg)	10.9±0.6	11.6±0.9	17.9±2.3	25.4±2.9*
ESV (µl)	10.2±0.5	11.3±0.4	21.6±0.9*	29.2±2.2#
EDV (µl)	27±0.7	26.8±1	34.2±0.6*	37.9±1.7#
Systolic function				
dP/dt max (mmHg/s)	10279±415	9942±538	8107±265*	6664±567#
Ea (mmHg/µl)	5.6±0.1	6.4±0.4	10.8±0.8*	14.6±0.9#
EF(%)	66.7±1.2	61.8±1.5	41.3±2.7*	27.9±1.7#
CO (µl/min)	9356±211	8157±438	6655±398*	4957±224#
Stroke volume (µl)	19.5±0.3	17.8±1.0	14.5±1.0*	10.8±0.4*
Diastolic function				
dP/dt min (mmHg/s)	−9340±422	−9308±286	−7874±419*	−5698±519#
Tau_w(ms)	7.4±0.2	7.4±0.4	9.2±0.4	12.3±1.4#

HR, heart rate; ESP, end-systolic pressure; EDP, end-diastolic pressure; ESV, end-systolic volume; EDV, end-diastolic volume; Ea, arterial elastance; EF, ejection fraction; CO, cardiac output; dp/dtmax, maximal rate of pressure development; dp/dtmin, maximal rate of pressure decay; Tau_w, time constant of isovolumic pressure decay.

*P<0.05 vs WT/sham.

# *P*<0.05 vs WT/AB after AB.

### ATF3 deficiency augments myocyte hypertrophy *in vitro*

**Figure 3:**
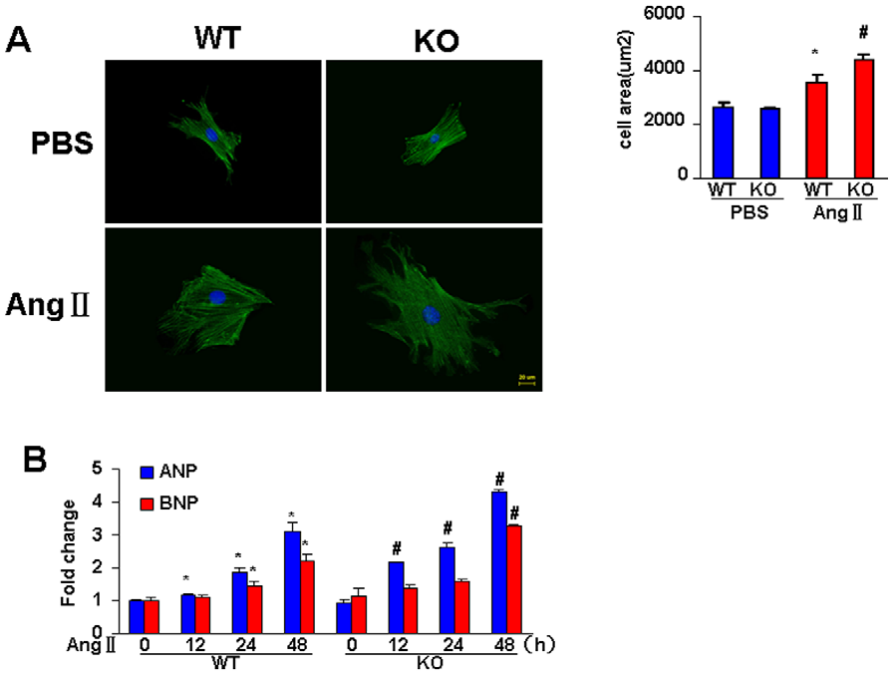
ATF3 deficiency augments myocyte hypertrophy *in vitro*. A, Effect of ATF3 deficiency on the enlargement of myocyte induced by Ang II (1 µM for 48 h). B, RT-PCR analysis of the mRNA levels of ANP and BNP induced by Ang II at the time points indicated. *P<0.05 vs WT group at the 0 time point. # *P*<0.05 vs WT group at the same time point.

To further confirm the effect of ATF3 on cardiac hypertrophy, we used an *in vitro* model with Ang II (1 µM) in cultured neonatal mouse cardiomyocytes. After stimulation with Ang II, myocytes from KO mice showed enlarged cell surface area compared to those from WT mice (Figure 3A). Moreover, RT-PCR demonstrated that ATF3 deficiency markedly enhanced the induction of ANP and BNP mRNA expression by Ang II (Figure 3B). These findings indicated that ATF3 deficiency promotes cardiac hypertrophy *in vitro*.

### Effects of ATF3 on MEK-ERK1/2 and JNK signaling

**Figure 4:**
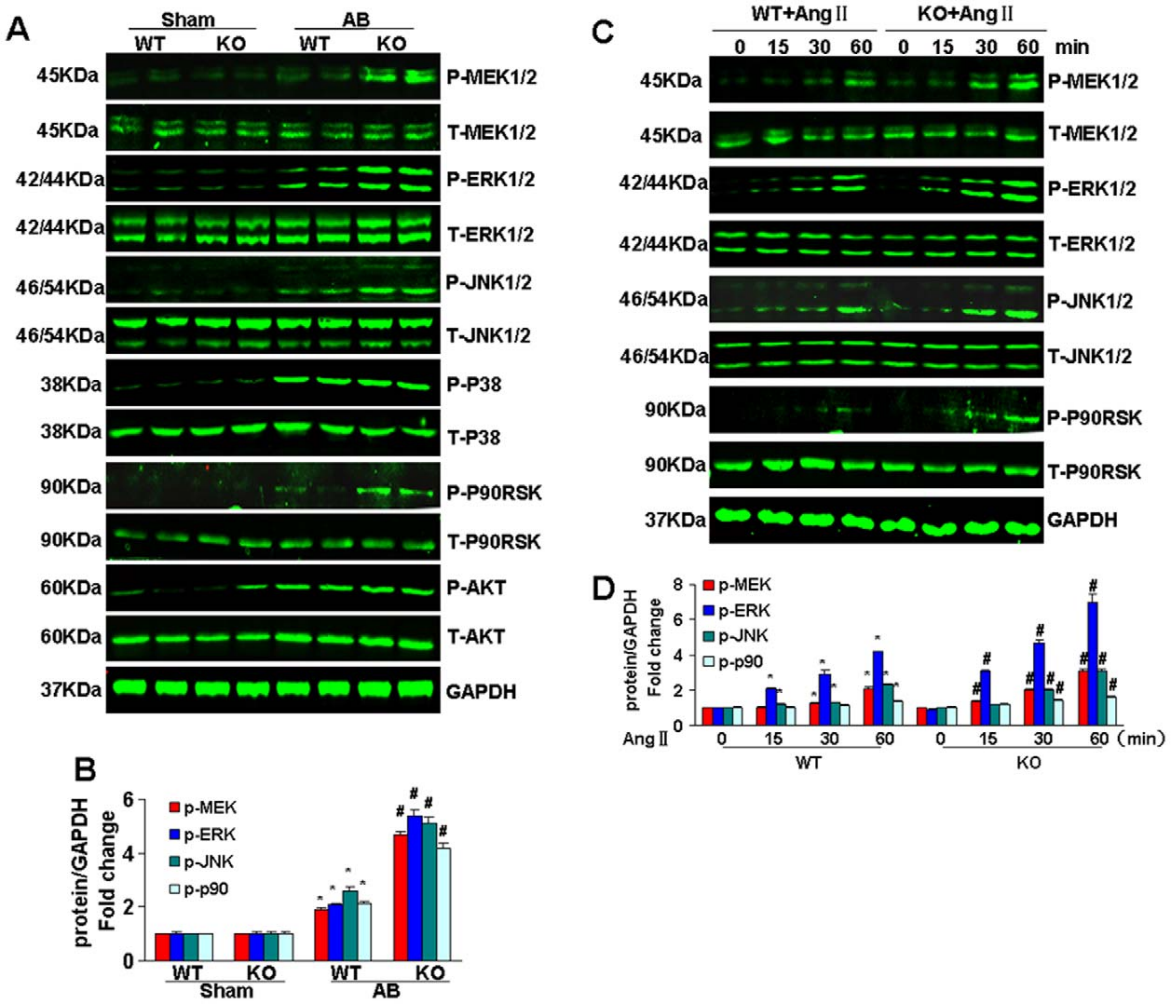
Effects of ATF3 on the ERK and JNK signaling pathways. (A and B) The levels of total and phosphorylated MEK1/2, ERK1/2, JNK, P38, P90RSK and AKT in the heart tissues of mice in the indicated groups (n = 6). A, Representative blots. B, Quantitative results. *P<0.05 vs WT/sham. # *P*<0.05 vs WT/AB after AB. (C and D) The levels of total and phosphorylated MEK1/2, ERK1/2, JNK and p90RSK in cardiac myocytes treated with Ang II at the indicated time points. C, Representative blots. D, Quantitative results. *P<0.05 vs WT group at the 0 time point. # *P*<0.05 vs WT group at the same time point.

To examine the molecular mechanism by which ATF3 affects the hypertrophic response, we investigated the activation of MAPK in KO and WT heart induced by pressure overload. Our data indicated that MEK-ERK1/2, JNK and p38 were significantly activated in AB mice and that ATF3 deficiency enhanced the phosphorylation of MEK-ERK1/2 and JNK. However, we did not find an difference in phosphorylated p38 levels between KO and WT mice. Furthermore, we examined the activation of 90-kDa ribosomal S6 kinase (p90RSK), a downstream effector of ERK1/2, and found that ATF3 deficiency amplified the phosphorylation of p90RSK in response to AB. Akt is another important signaling molecule involved in cardiac hypertrophy, and our data showed that Akt activation was increased in AB mice, although there was no striking difference between the KO and WT group (Figure 4A, B). *In vitro* data confirmed that the activation of MEK-ERK1/2, JNK and p90RSK in myocytes from KO mice was greater compared to WT mice in response to AngII (Figure 4C, D).

### ATF3 deficiency exacerbates the fibrotic response induced by pressure overload

**Figure 5:**
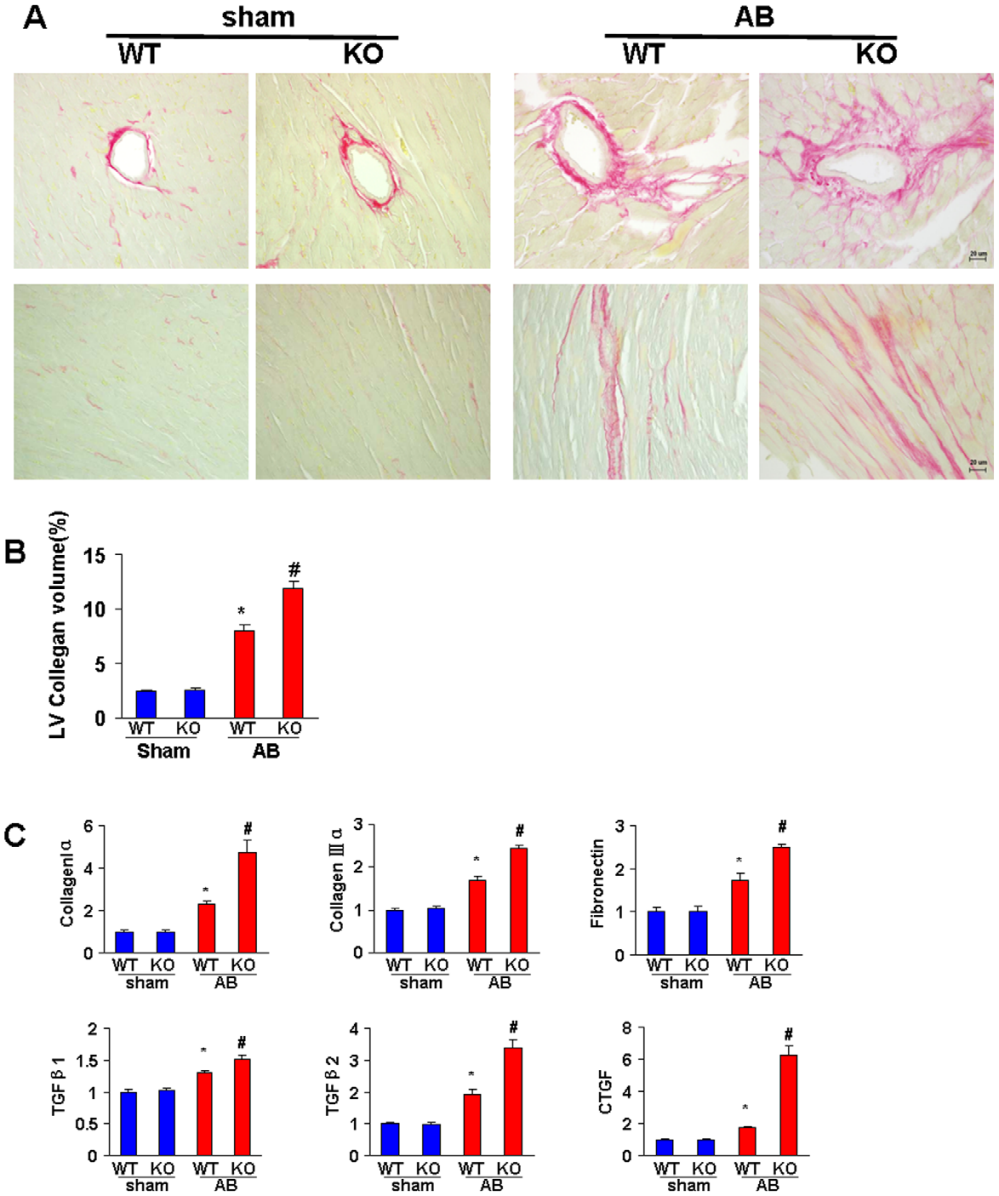
ATF3 deficiency exacerbates the fibrotic response induced by pressure overload. A, Histological sections of the left ventricle were stained for picrosirius red for the indicated groups. B, Fibrotic areas from histological sections were quantified using an image-analyzing system. C, The mRNA expression of collagen I, collagen III, fibronectin, TGF-β1, TGF-β2, and CTGF in the myocardium were obtained from indicated groups using RT-PCR analysis. *P<0.05 vs WT/sham. # *P*<0.05 vs WT/AB after AB.

Fibrosis is an important feature of developing pathological cardiac hypertrophy. To detect fibrosis, heart sections were stained with PSR. Striking perivascular and interstitial fibrosis was observed in the WT mice in response to AB, and the extent of cardiac fibrosis was remarkably increased in the KO mice (Figure 5A, B). No significant difference in fibrosis was detected between the WT and KO mice after the sham operation.

To further elucidate the effect of ATF3 on collagen synthesis, we examined the mRNA expression of connective tissue growth factor (CTGF), transforming growth factor (TGF)-β1, TGF-β2, collagen I, collagen III, and fibronectin, which are responsible for cardiac fibrosis. Our data showed that ATF3 deficiency enhanced the increase of CTGF, TGF-β1, TGF-β2, collagen I, collagen III, and fibronectin expression 4 weeks after AB (Figure 5C).

## Discussion

Previous studies have proven that the ATF/CREB family plays an important role in the cardiovascular system [11], [12], [13], [14], [19]. In this study, we demonstrated that ATF3 deficiency worsened cardiac hypertrophy induced by pressure overload *in vivo* and by Ang II *in vitro*. The effects of ATF3 on cardiac hypertrophy, dysfunction and fibrosis are probably mediated by negative feedback to the ERK and JNK pathways and regulation of pro-fibrotic cytokines. These results suggest that ATF3 may play a protective role in pathological hypertrophy in the heart and may be an effective therapeutic target for cardiac hypertrophy and heart failure.

ATF3, a member of the ATF/CREB family, contains the bZIP element and plays a role as either a repressor or activator of transcription via forming homodimers or heterodimers with other bZIP-containing proteins such as ATF2 and c-Jun [4]. ATF3 is an adaptive-response gene and is induced by various environmental stresses, including injury, ischemia, ischemia/reperfusion, chemical toxin, cytokines, chemokines, and growth factors/hormones [5], [7], [8]. Previous studies have shown increased ATF3 expression in mice injected with Ang II, isoproterenol or phenylephrine [14], [20]. We demonstrated that high ATF3 expression was not only in response to aortic banding in mice but also in the hearts of patients with DCM, which suggests that ATF3 is involved in the development of cardiac hypertrophy. Mice with cardiac-specific over-expression of ATF3 exhibited atrial enlargement and cardiac hypertrophy in a previous report [13]; however, our results demonstrated that ATF3 deficiency promotes pathological hypertrophy. It seems obscure that the role of ATF3 in cardiac hypertrophy is whether detrimental or protective. The first possibility for the discrepancy is that the transgenic mice have a persistent expression of ATF3. Previous study showed that adenovirus-mediated expression of ATF3 could inhibit doxorubicin-induced cardiomyocytes apoptosis [12], which indicated that transient expression of ATF3 may be protective on cardiomyocytes. Loss of ATF3 may impair the resistant ability of heart under stress. Secondly, the expression level of ATF3 in the transgenic mice may be too high for the homeostasis. Thirdly, ATF3 have dual roles in the regulation of transcription: as either a repressor or activator [4]. Therefore, it is likely that ATF3 is necessary in the defense against pathological stress in the heart, and its up-regulation may be protective in the progression of cardiac hypertrophy. However, persistent overexpressed ATF3 may play as a transcriptional activator rather than repressor of pro-hypertrophic genes, which is detrimental to the heart, indicating that it is important to regulate the level and duration of ATF3 expression to resist hypertrophic stress.

Fibrosis, which is an integral feature of cardiac remodeling, is a disproportionate accumulation of fibrillar collagen leading to expansion of the extracellular matrix (ECM) and cardiac dysfunction [21]. Myocardial fibrosis following pressure overload is associated with increased accumulation of type I and III collagen within the adventitia of coronary arteries (perivascular fibrosis), which progressively extends into the neighboring interstitial spaces (interstitial fibrosis) [22]. We found enhanced deposition of collagen in ATF3-KO mice after AB and demonstrated that ATF3 deficiency promotes collagen synthesis, with up-regulated mRNA levels of collagen I and III. TGFβ and CTGF are two major extracellular signals that promote fibrosis in the hypertrophic heart [23], [24], and our study showed increased mRNA expression of these cytokines in ATF3-KO mice after AB. These results suggest that ATF3 could protect against cardiac remodeling through regulation of pro-fibrotic cytokines and collagen content.

The molecular mechanisms by which ATF3 affects cardiac remodeling remain unclear. Recent evidence has suggested that the MAPK cascade is involved in cardiac hypertrophy and fibrosis [25], [26]. The MAPK cascade is initiated in cardiac myocytes by various stress stimuli. After activation, downstream p38, JNKs, and ERKs each phosphorylate a wide array of intracellular targets, including numerous transcription factors, resulting in the reprogramming of cardiac gene expression [27]. ATF3 expression is also regulated by the MAPK cascades [28], [29]. ERK pathway is necessary in serum-induced ATF3 expression, which was evidenced by that the specific MEK1 kinase inhibitor PD98059 almost completely abolished the serum induction of ATF3 by suppressing the phosphorylation of ERK [30]. And, the activation of the ERK is essential for ATF3 induction in the left atrium following acute angiotensin II stimulation [14]. JNK pathway is involved in the induction of ATF3 by acute hypoxia and homocysteine in endothelial cells, which was confirmed by using specific inhibitor or expressing dominant negative upstream moleculars of JNK [10], [31]. Furthermore, ATF3 is also activated by JNK in doxorubicin-treated neonatal rat cardiomyocytes [12]. On the other hand, ATF3 could suppress tumorigenesis stimulated by Ras [32] which is upstream of MEK-ERK1/2 and JNK, and prevent JNK-induced neuronal death [33]. Results of these studies indicated the interaction between ATF3 and MEK-ERK1/2 and JNK. In our study, we found that pressure overload induced the activation of ERK1/2, JNK and p38, as well as MEK1/2, the upstream activator of ERK1/2. We then demonstrated that ATF3 deficiency enhanced the increased phosphorylation of MEK-ERK1/2 and JNK after AB but did not affect p38 phosphorylation. P90RSK, a downstream effector of ERK1/2, regulates a number of transcription factors and interacts with other kinases involved in hypertrophic response [34], [35]. Our results showed that ATF3 deficiency increased the phosphorylation of p90RSK in response to AB. Akt is another pathway that play crucial role in cardiac hypertrophy [36]. A recent study reported that ATF3 affected mast cells development and function via the Akt pathway [37]; however, we did not detect the influence of ATF3 on Akt phosphorylation. Previous studies have described ATF3 as a “hub” of the cellular adaptive-response network [8], indicating the complex role of ATF3 in diseases. Our results demonstrated that ATF3 provides negative feedback to the ERK and JNK pathways to modulate cardiac remodeling, providing more clues into the functional network of ATF3. However, further experiments are needed to determine the molecular mechanism by which ATF3 regulates ERK and JNK pathways.

In conclusion, this study reveals a previously unknown effect of ATF3 on cardiac hypertrophy, dysfunction and fibrosis in response to hypertrophic stimuli by the negative feedback to the ERK and JNK pathways and regulation of pro-fibrotic cytokines and collagen content. Our study provides new insight into the pathogenesis of cardiac remodeling and may have important implications for the development of strategies for the treatment of cardiac hypertrophy and heart failure through targeting ATF3.
